# A high-resolution palaeoenvironmental record from carbonate deposits in the Roman aqueduct of Patara, SW Turkey, from the time of Nero

**DOI:** 10.1038/srep28704

**Published:** 2016-06-30

**Authors:** Cornelis Passchier, Gül Sürmelihindi, Christoph Spötl

**Affiliations:** 1 Department of Earth Sciences, University of Mainz, Mainz, Germany; 2 Department of Geology, University of Innsbruck, Innsbruck, Austria

## Abstract

An inscription on the supporting wall of the inverted siphon of the aqueduct of the ancient Roman city of Patara, SW Turkey, explains how the wall collapsed during an earthquake and was subsequently restored. Carbonate deposits formed inside the aqueduct channel show cyclic stable isotope changes representing 17 years of deposition. This sequence, together with the text of the inscription, allows dating the earthquake to 68 AD and the original inauguration of the aqueduct to the winter of 51/52 AD. Thus, the carbonate deposits represent a high-resolution record of palaeotemperature and precipitation for SW Turkey covering the complete reign of the Emperor Nero. The period shows a cooling and drying trend after an initial warm and more humid period, interrupted by a few anomalous years. These 2 cm of calcite highlight the significance of carbonate deposits in ancient water supply systems as a high-resolution archive for palaeoclimate, palaeoseismology and archaeology.

Carbonate deposits formed in ancient water structures can provide a wealth of historical information on environmental change, natural disasters and human interference^1^,^2^,^3^. During the Roman Empire, more than 1700 long-distance supply lines and thousands of smaller-scale aqueducts were built to deliver bathing and drinking water to cities, mines, villas and thermae^4^,^5^,^6^. Since most of these structures were fed by perennial karst springs, carbonate deposits commonly precipitated in these supply channels and recorded variations in water temperature, evaporation, discharge and composition, as well as environmental and/or human interference with these fragile infrastructures. Since most ancient water supplies were built as pipelines or square masonry channels of regular dimensions and measurable slope where water was kept running for decades, the carbonate deposits formed therein represent a – largely untapped – archive of palaeoclimate, palaeohydrology, palaeoseismology and archaeology^7^,^8^,^9^,^10^. The deposits are specifically useful as a high-resolution palaeotemperature record, because they are commonly annually banded and may record seasonal air temperature fluctuations^1^,^2^.

## Delikkemer Siphon of the Patara Aqueduct

**Figure 1:**
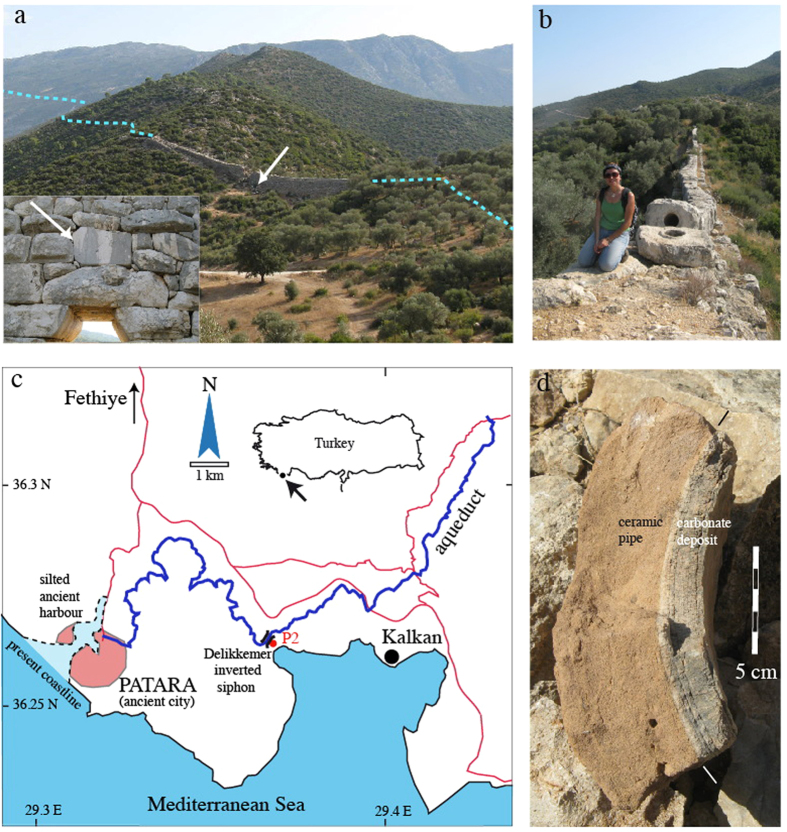
General background. (**a**) Inverted siphon of the Patara aqueduct line, named Delikkemer. The trace of the aqueduct channel is shown as a blue dotted line, water flow from left to right. Location of the inscription is shown by the white arrow. Photograph looking south. Inset: ashlar carrying the inscription (arrow); (**b**) stone blocks of the restored Vespasianic Roman inverted siphon; (**c**) map of the aqueduct showing the location of the inverted siphon and sample P2. Adobe illustrator CS3 v13.0.2; coastline and roads from GoogleEarth Data SIO, NOAA, U.S. Navy, NGA, GEBCO. Image © 2016 Terrametrics, © 2016 Basarsoft, Image © 2016 Digitalglobe. Aqueduct trace our observations; (**d**) thick-walled (4.8–5 cm) ceramic pipe fragment with attached carbonate deposit similar to that from which sample P2 was derived.

We examined carbonate deposits from the aqueduct of the Roman harbour city of Patara, SW Turkey, which consisted of ceramic pipes and masonry channel sections with a combined length of 23 km and a vertical descent of 612 m (Fig. 1c)^2^,^11^,^12^. The structures were built on top of bedrock, and are only locally buried at shallow depth, permitting significant cooling in winter and warming in summer^2^. The aqueduct was fed by a karst spring and calcite deposits formed in the channel^2^. The water in the aqueduct was running by gravity and at atmospheric pressure, except for a 200 m-long and 18 m-deep inverted siphon, known as Delikkemer^2^,^11^,^12^, where the water crossed a 20 m-deep valley in a closed pipe under pressure (Fig. 1a,b). This siphon presently consists of a row of hollowed limestone blocks placed on a polygonal wall. On both sides of the wall, an identical inscription was placed which reads^13^,^14^: 


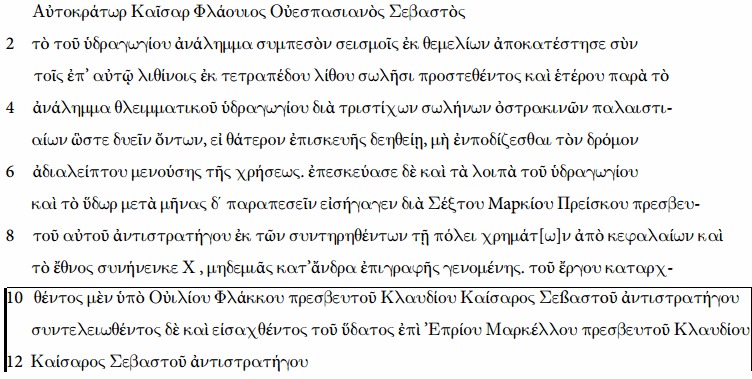


“After the earthquake had brought the wall of the aqueduct to collapse, the Emperor *Caesar Flavius Vespasian Augustus* ordered to rebuild it from the bottom up, together with the pipeline of hewn stone that runs on her, and additionally a pressure line of clay pipes one handbreadth wide in three rows along the wall, such that the course of the water is not interrupted when the line needs maintenance, because there are two lines. He also overhauled the rest of the aqueduct and caused the water, that had spilled for four months, to be brought (into the city) by *Sextus Marcius Priscus*, his legate of propraetorian rank. (All was paid) without charging the taxpayer by any special payments, from the funds that were kept back for the city of the poll tax, and the federal government also contributed… denarii. *Vilius Flaccus,* legate of *Claudius Caesar Augustus* of propraetorian rank, had the construction of the aqueduct started, and it was completed and water brought into the city under *Eprius Marcellus,* legate of *Claudius Caesar Augustus* of propraetorian rank”^13^,^14^,^15^ (translation from German^14^ by the authors).

It is unusual that such detailed technical information on aqueduct construction and repair is available for an ancient water supply line. Carbonate deposits from the siphon were therefore analysed to see if they confirm the text of the ancient inscription.

Taking into account the known periods of the governorship of the legates mentioned in the inscription, the text implies that construction of the aqueduct started shortly after the annexation of Lycia during the reign of Emperor *Claudius,* in 48–49 AD by Governor *Vilius Flaccus,* and was finished in 50–55 AD by Governor *Eprius Marcellus*. It also reports that the aqueduct was destroyed during an earthquake between 65 and 72 AD, because it was restored during the reign of Emperor *Vespasian*, who came to power in November 69 AD^13^,^14^, by his Governor *Sextus Marcius Priscus*, who ruled until 70 or 71 AD^13^,^14^. The only historically known earthquake in the area during this time period is that of spring 68 AD, shortly before the death of Emperor *Nero* in June of that year^13^,^14^. The aqueduct mentioned in the inscription therefore encompasses a historically important period, the entire reign of Emperor *Nero*.

Fragments of ceramic pipes of different diameter were found at the foot of the Delikkemer inverted siphon, including remains of the “pipes one handbreadth wide” (one palmus, 74 mm), which are mentioned in the inscription^4^. Several fragments, however, are from larger diameter (30 cm) ceramic pipes with 4.5–5.0 cm thick walls (Fig. 1d). The thickness of the pipe walls, and the fact that carbonate deposits in them are of nearly constant thickness around the pipe suggest that these pipes ran full, and were part of a siphon structure. Fragments of these pipes with carbonate still attached were built into the wall that supports the stone blocks of the present siphon, testifying that these fragments were part of a water pipe that predates the restoration of the aqueduct. This observation, the thickness of the pipes, and the presence of carbonate deposits suggest that the original *Claudian* inverted siphon, which was destroyed by the mentioned earthquake of 68 AD, was made of ceramic pipes, which were replaced by hollowed limestone blocks during the restoration under *Vespasian*.

### Sample P2

**Figure 2:**
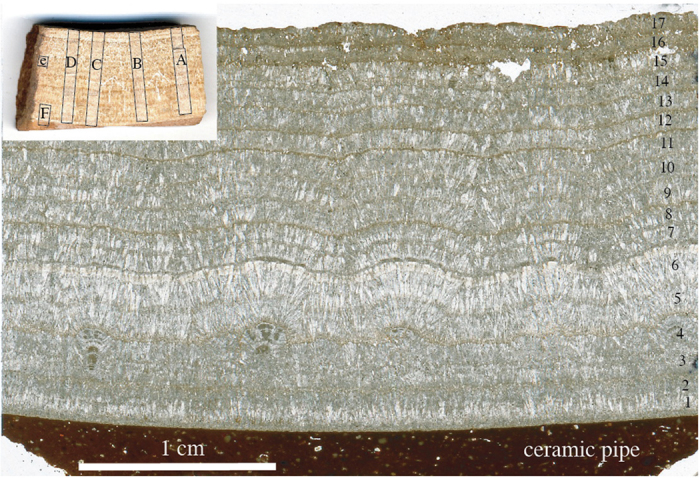
Investigated sample. Thin section of sample P2, showing a regular alternation of thick microsparite and thin micrite layers. Notice the slight curvature of the sample, which followed the large diameter of the siphon pipe. Layers are numbered corresponding to peaks in stable isotopes (Fig. 3). Inset: the original sample with micromill traces of Fig. 3.

**Figure 3:**
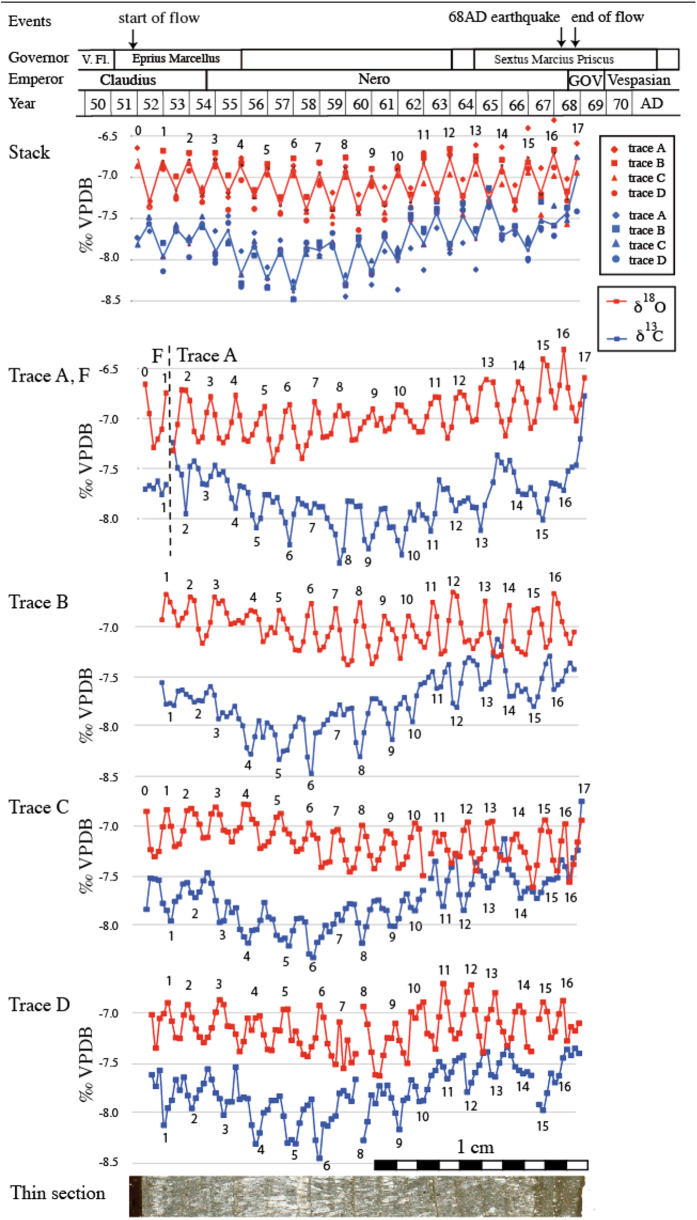
Stable isotope data obtained from sample P2. A stack was constructed using maximum values of four parallel micromill traces. Maxima are numbered, and corresponding years and relevant periods are shown at the top. Trace A is incomplete at the bottom, and extended by trace F milled near the bottom of the sample in another location. The micromill traces are shown in Fig. 2 inset. GOV – Galba, Otho, Vitellius, year of the Four Emperors. V. Fl – Vilius Flaccus. Arrows indicate major events mentioned in the text.

A 21 mm-thick complete section of carbonate deposits from one of these ceramic pipes, P2, was briefly investigated in earlier studies^1^,^2^ and in more detail in this study (Figs 1d and 2) to see if the inscription text could be confirmed, and if the deposits contain paleoenvironmental information. The pipe of P2 (Fig. 1d) had an inner diameter of 29–32 cm and a wall thickness of 4.8 cm. P2 is more dense and coarser crystalline than calcite crusts from the open masonry channel section of the same aqueduct which are more micritic, probably due to the higher flow speed of the water in the siphon pipe precluding biofilm growth^1^,^2^. The calcite is regularly laminated with an alternation of microsparite and micrite except for the top 2 mm, which are relatively more porous and micritic^2^. Microspar crystals grew by growth competition, forming bundles of crystals up to 2 mm long (Fig. 2). These bundles are abruptly truncated by a thin micrite layer, forming microsparite-micrite cycles (Fig. 2). 15–19 couplets of microsparite-micrite laminae are present in this sample. The regularity of these couplets strongly suggests they are annually laminated layers. This was confirmed by observations of other samples from the Patara aqueduct as well as the Aspendos aqueduct^1^,^2^, also in southern Turkey, which showed that microsparite formed in winter during high discharge and micrite in summer during reduced water flow, possibly related to enhanced biogenic activity due to reduced water flow and increased water temperature. The central part of trace A in Fig. 3 was previously^1^,^2^ used, in combination with water data from the source, to demonstrate a close relationship between the stable isotope data and the crystal fabric. P2 is the furthest downstream sample on an 11 km-long stretch of the aqueduct channel and therefore has the most pronounced stable isotope cyclicity of all samples of this aqueduct^1^,^2^.

### Stable isotope results

While petrographic laminae are indistinct in parts of the sample and cannot be used to reliably count years, stable isotopes were analysed at sufficiently high resolution to provide a clear annual pattern, which was replicated in four parallel profiles milled perpendicularly to the layering (Fig. 3). δ^18^O and δ^13^C curves show a regular, mirror-like cyclicity. Individual laminae are not flat, which hampers correlation of the isotope signal with petrography, but micrite laminae usually correlate with minima in δ^18^O, and microsparite with maxima, as observed in other samples of the Patara aqueduct^2^. The cyclic changes of δ^18^O could be the result of regular changes in the isotopic composition of the spring water, degree of evaporation, or water temperature that drives isotope fractionation during calcite precipitation. The modern spring water shows only a very small intra-annual variation (−7.3 to −7.1‰ VSMOW)^2^ and evaporation is considered insufficient in a closed and covered aqueduct to explain the observed cyclicity^2^. We therefore attribute the δ^18^O cyclicity to temperature-dependent isotope fractionation reflecting the seasonal temperature gradient in the aqueduct water, consistent with previous studies^1^,^2^. Low atmospheric temperatures during winter lead to low water temperatures in the aqueduct and hence to δ^18^O maxima of the calcite (larger fractionation at isotope equilibrium) and vice versa. The small amplitude in δ^18^O of 0.3 to 0.7‰ is consistent with a strong buffering of seasonal air temperature swings by both the flowing water and the stone and cement of the covered aqueduct channel. The annual temperature of the aqueduct water is (a) a function of the spring temperature, which today is highly invariant (12.4–12.7 °C), (b) the water volume and flow speed in the channel, which can be calculated from the channel slope and the height of deposits on the channel walls, and (c) the heat flow from the aqueduct structure to the water in channel, upstream from the sampling site of P2. A precise calculation of this heat flow depends mainly on the nature of the channel cover. A first-order estimate was made^2^, but more detailed archaeological work is needed to establish the thickness and nature of the cover along the upstream part of the aqueduct. Once such data are available, annual water temperature fluctuations in the channel, as constrained by calcite δ^18^O values, can be used to assess annual air temperature fluctuations in Patara.

## Discussion

**Figure 4:**
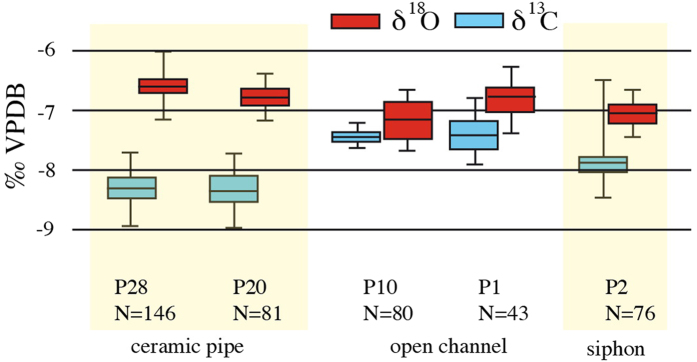
Box and whisker diagram of δ^18^O and δ^13^C values of selected calcite deposits from the Patara aqueduct. The yellow domains mark samples from closed pipes, the white domain encompasses samples from open, slab-covered masonry channels. Sample P2 is shown at right. δ^13^C values are generally higher in the open channel sections, probably due to higher degassing. Modified after^2^.

The antithetic pattern of the δ^13^C curve with respect to δ^18^O is attributed to biofilm growth and to water level and temperature-induced variations in the rate of CO_2_ degassing in the open channel upstream of the siphon^2^. An unusual feature of the δ^13^C curve is the sharp increase at the end of the sequence in the last cycle of the deposit, visible in traces A and C (Fig. 3); this youngest layer was not preserved in the other two profiles due to abrasion of the fragile deposit. We checked if this rise in δ^13^C might be a weathering phenomenon near the surface of the deposit and analysed the equally weathered and broken left-hand side of the sample (Fig. 2 inset, trace e), but no increase in δ^13^C was observed there, suggesting that the final rise in δ^13^C is a locally preserved pristine geochemical feature. A comparison of the mean δ^13^C and δ^18^O values along the course of aqueducts given in an earlier study^2^ shows that open channel deposits show higher δ^13^C values than those in closed pipes (Fig. 4)^2^. We attribute this to reduced degassing in closed pipes, leading to lower values. The large increase in δ^13^C at the end of the sequence to approximately −6.7‰ approaches δ^13^C values typical of open channel deposits (Fig. 4). This rise is unprecedented in the 17 year-history of this aqueduct and likely reflects a sudden increase in the degassing rate, probably caused by damage of the pipes or the cover of the aqueduct, such as would be expected during a major earthquake.

The youngest deposits formed during autumn or winter (Fig. 2). If the cessation of water flow in the investigated siphon sample was indeed due to an earthquake in the spring or early summer of 68 AD^13^,^14^, water must have continued to flow for some months before deposition terminated, in agreement with the inscription text^14^,^15^. The sudden rise in δ^13^C in this period is consistent with enhanced degassing due to extended damage to the aqueduct.

The exact date of the earthquake allows us to reconstruct the inauguration year of the aqueduct. The δ^18^O sequence starts with a high value, i.e. during winter. This strongly suggests that the aqueduct started operating in the winter of 51–52 AD, which is the end of the first year of the governorship of the *Claudian* legate *Eprius Marcellus*, as stated in the inscription. Building of the aqueduct therefore took 1–3 years, since *Vilius Flaccus* started his governorship in 48 AD. The annually laminated carbonate deposits clearly confirm the inscription text, and in addition allow a more precise dating of the start of aqueduct operation. The 21 mm-thick calcite deposits encompass the last years of the reign of Emperor *Claudius* and the entire reign of Emperor *Nero* (54–68 AD), and its isotopic composition provides seasonally resolved environmental information for this period for SW Turkey^16^,^17^. The four profiles show small isotopic differences, reflecting the uneven nature of the laminae, and each section comprises slightly different parts of the stratigraphy. We therefore established an isotope stack using the maximum and minimum values of each cycle in each trace (Fig. 3). As detailed above, the oxygen isotopic composition primarily reflects changes in air temperature via the isotopic fractionation during calcite precipitation, buffered by the thermal inertia of the aqueduct structure and the flowing water, in a similar manner as in cave settings.

The stacked absolute isotopic values of each year reflect the extent to which individual summers and winters were warmer or colder than others. The stack also shows a multi-annual trend in the stable isotope composition over the 17 year-period of aqueduct operation (Fig. 3). Given that there is no evidence of changes in the maintenance of the aqueduct (neither from textural sources nor from the stratigraphy of the deposit) we attribute these trends to decadal-scale changes in air temperature and precipitation.

The δ^18^O data therefore suggest a gradual increase in summer and winter temperatures during the first decade, followed by a cooling, reflected in a slight decline and a subsequent rise of the curve (Fig. 3). This observation is consistent with other paleoclimate data for the Roman period^16^,^17^. The period 27–56 AD was particularly warm and followed by a period of cooling probably due to decreasing solar activity^16^,^17^. Our data clearly show a gradual increase in δ^18^O after 59 AD, especially in the minima of the curve, suggesting cooler summers. The years 52, 58, 59, 60, and 66 AD stick out as particularly warm summers, while 53, 54, 55 and 65 AD were the opposite. 63/64, 64/65 and 67/68 were rather cold winters, whereas the winters of 58/59, 60/61, 65/66 were mild.

The annual cyclicity of δ^13^C reflects seasonal variations in the degassing rate with less degassing occurring during winter, probably coinciding with a high water level (discharge). From 51 to 57 AD, the δ^13^C curve shows a decline followed by gradual increase afterwards. This could be due to a first increasing and then decreasing discharge of the aqueduct, probably driven by long-term changes in rainfall. The winters of 58/59 and 65/66 are characterised by high δ^13^C values, probably reflecting enhances degassing due to reduced discharge in relatively dry and mild winters. 57/58, 59/60 and 66/67 were probably rather wet winters (reduced degassing and hence rather low δ^13^C values). Rather thick microsparite laminae were deposited during these winters.

Of interest is also the slight divergence and subsequent strong convergence of δ^18^O and δ^13^C curves between 51 and 68 AD. The initial warming trend coincided with an increase in precipitation, while the cooling trend commencing in the second half of Emperor *Nero*’s reign was accompanied by an overall decrease in rainfall, consistent with proxy data for the eastern Mediterranean^18^.

The Patara aqueduct can be seen in the light of overall Imperial strategy, following a period of repeated famine in Rome^19^,^20^ in the reign of the Emperor *Claudius*. The aqueduct was built after the annexation of Lycia, when Patara became a centre of maritime trade and a vital link in the Egyptian grain supply of Rome. It is therefore fitting that this same aqueduct can now provide data on the actual climate conditions that ruled the fate of the Roman Empire.

## Conclusions

- Carbonate deposits in Roman aqueducts are a largely untapped high-resolution archive for palaeoclimate, archaeology and palaeoseismology.
- An inverted siphon in the Roman aqueduct of Patara was destroyed by a major earthquake in 68 AD under the reign of Emperor *Nero*.
- Annual layering of carbonate deposits in an inverted siphon supported by stable isotope data indicates 17 years of aqueduct operation. This allows dating the inauguration of the aqueduct to the winter of 51/52 AD, in accordance with archaeological data.
- High-resolution oxygen and carbon stable isotope data reflect intra- and interannual air and water temperature variations, and variations in precipitation. Although qualitative, they allow to establish a temperature and precipitation record for SW Turkey during the reign of Emperor *Nero*. Such data, if replicated by other aqueducts and/or other equally highly resolved proxy data e.g. from speleothems or dendrochronology, are of great value to refine our understanding of the climate in the Mediterranean region for the Roman Era.

## Materials and Methods

Sample P2 was cut using a thin diamond saw and two mirror-image slabs were used to produce a polished thin section (Fig. 2) and a polished sample (Fig. 2-inset). The microstructure was investigated by transmitted-light microscopy. The polished slab was micromilled at 0.2 mm intervals in four parallel traverses (5 mm wide) perpenticular to the lamination. The least porous and well-laminated parts of the sample were chosen for the micromill traverses. Sample powders were analyzed at the University of Innsbruck using a semi-automated device (Gasbench II) linked to a ThermoFisher Delta^plus^XL isotope ratio mass spectrometer. Isotope values are reported on the VPDB scale and long-term precision is better than 0.1‰ for both δ^13^C and δ^18^O^21^. Standardization of the mass spectrometer was achieved using international calcite standards (including NBS18, NBS19, CO1, CO8). The stack in Fig. 3 was produced using the extreme values of corresponding cycles in each of the four measured traverses and connecting the mean values.

## Additional Information

**How to cite this article**: Passchier, C. *et al*. A high-resolution palaeoenvironmental record from carbonate deposits in the Roman aqueduct of Patara, SW Turkey, from the time of Nero. *Sci. Rep.* **6**, 28704; doi: 10.1038/srep28704 (2016).
